# Exploring directional and fluctuating asymmetry in the human palate during growth

**DOI:** 10.1002/ajpa.24293

**Published:** 2021-05-11

**Authors:** Gregorio Oxilia, Jessica C. Menghi Sartorio, Eugenio Bortolini, Giulia Zampirolo, Andrea Papini, Marco Boggioni, Sergio Martini, Filippo Marciani, Simona Arrighi, Carla Figus, Giulia Marciani, Matteo Romandini, Sara Silvestrini, Maria Elena Pedrosi, Tommaso Mori, Alessandro Riga, Ottmar Kullmer, Rachel Sarig, Luca Fiorenza, Melchiore Giganti, Rita Sorrentino, Maria Giovanna Belcastro, Jacopo Moggi Cecchi, Stefano Benazzi

**Affiliations:** ^1^ Department of Cultural Heritage University of Bologna Ravenna Italy; ^2^ Department of Enterprise Engineering “Mario Lucertini” – Tor Vergata University Rome Italy; ^3^ Section for Evolutionary Genomics Øster Farimagsgade University of Copenhagen København Denmark; ^4^ Dentist's Surgery via Walter Tobagi 35 Prato 59100 Italy; ^5^ Dentist's Surgery via D'Andrade 34/207 Genoa Sestri Ponente 16154 Italy; ^6^ Dental Lab Technician via Milani, 1 Verona 37124 Italy; ^7^ Dentist's Surgery, Studio Dentistico Marciani Dr. Filippo Via Romagnoli, 14 Lanciano 66034 Italy; ^8^ Department of Biological, Geological and Environmental Sciences– BiGeA University of Bologna Bologna Italy; ^9^ Department of Biology University of Florence Florence Italy; ^10^ Senckenberg Research Institute Senckenberganlage 25 Frankfurt am Main 60325 Germany; ^11^ Department of Paleobiology and Environment Institute of Ecology, Evolution, and Diversity, Johann Wolfgang Goethe University Frankfurt Germany; ^12^ Department of Oral biology The Goldschleger School of Dental Medicine and the Dan David Center for Human Evolution, the Sackler Faculty of Medicine, Tel‐Aviv University Tel‐Aviv Israel; ^13^ Monash Biomedicine Discovery Institute, Department of Anatomy and Developmental Biology Monash University Melbourne Victoria Australia; ^14^ Department of Morphology, Surgery and Experimental Medicine University of Ferrara Ferrara Italy; ^15^ Radiology University Unit University Hospital Ferrara Italy; ^16^ Department of Human Evolution Max Planck Institute for Evolutionary Anthropology Leipzig Germany

**Keywords:** directional asymmetry, fluctuating asymmetry, ontogeny, palatal arch

## Abstract

**Objectives:**

Palate morphology is constantly changing throughout an individual's lifespan, yet its asymmetry during growth is still little understood. In this research, we focus on the study of palate morphology by using 3D geometric morphometric approaches to observe changes at different stages of life, and to quantify the impact of directional and fluctuating asymmetry on different areas at different growth stages.

**Materials and Methods:**

The sample consists of 183 individuals (1–72 years) from two identified human skeletal collections of 19th and early 20th Century Italian contexts. A 3D‐template of 41 (semi)landmarks was applied on digital palate models to observe morphological variation during growth.

**Results:**

Asymmetrical components of the morphological structure appears multidirectional on the entire palate surface in individuals <2 years old and become oriented (opposite bilateral direction) between 2 and 6 years of age. Specifically, directional asymmetry differentially impacts palate morphology at different stages of growth. Both the anterior and posterior palate are affected by mild alterations in the first year of life, while between 2 and 6 years asymmetry is segregated in the anterior area, and moderate asymmetry affects the entire palatal surface up to 12 years of age. Our results show that stability of the masticatory system seems to be reached around 13–35 years first by females and then males. From 36 years on both sexes show similar asymmetry on the anterior area. Regarding fluctuating asymmetry, inter‐individual variability is mostly visible up to 12 years of age, after which only directional trends can be clearly observed at a group level.

**Discussion:**

Morphological structure appears instable during the first year of life and acquires an opposite asymmetric bilateral direction between 2 and 6 years of age. This condition has been also documented in adults; when paired with vertical alteration, anterior/posterior asymmetry seems to characterize palate morphology, which is probably due to mechanical factors during the lifespan. Fluctuating asymmetry is predominant in the first period of life due to a plausible relationship with the strength of morphological instability of the masticatory system. Directional asymmetry, on the other hand, shows that the patterning of group‐level morphological change might be explained as a functional response to differential inputs (physiological forces, nutritive and non‐nutritive habits, para‐masticatory activity as well as the development of speech) in different growth stages. This research has implications with respect to medical and evolutionary fields. In medicine, palate morphology should be considered when planning orthodontic and surgical procedures as it could affect the outcome. As far as an evolutionary perspective is concerned the dominance of directional asymmetries in the masticatory system could provide information on dietary and cultural habits as well as pathological conditions in our ancestors.

## Graphical abstract

Directional and fluctuating asymmetry of human palate.

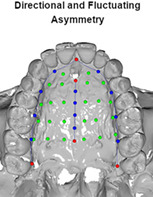

## 1 INTRODUCTION

Bilateral symmetry refers to body symmetry assessed with reference to a craniocaudal axis (sagittal plane) that affects the majority (>99%) of modern animals, including humans (Finnerty, 2005; Finnerty et al., 2004; Klingenberg et al., 2002; Mardia et al., 2000). Deflections from symmetry have been widely explored through quantitative methods (e.g., Auffray et al., 1996; Auffray et al., 1999; Bookstein, 1996; Kent & Mardia, 2001; Klingenberg & McIntyre, 1998; Mardia et al., 2000; Smith et al., 1997) establishing that deviations from perfect bilateral symmetry can occur when the individual has been subjected to anomalous developmental conditions as well as environmental and genetic stress (Auffray et al., 1999; Klingenberg et al., 2001; Klingenberg et al., 2002; Klingenberg & McIntyre, 1998; Klingenberg & Zaklan, 2000; Møller & Swaddle, 1997; Palmer & Strobeck, 1986; Parsons, 1992; Vazzana et al., 2018).

The degree of asymmetry in anatomy may indicate a genetic (Boder, 1953; Cassidy et al., 1998; Lundstrӧm, 1961; Melnik, 1992; Moreira et al., 2008; Wolpert et al., 2006), congenital or acquired pathological condition (Bishara et al., 1994) as well as abnormal habits, such as finger sucking (Lundstrӧm, 1961; Reid & Price, 1984; Yamaguchi & Sueishi, 2003). Knowledge of difference between “normal asymmetry” (Functional) and “pathologic asymmetry” (Para‐functional) is important for diagnosis of medical conditions.

Functional activities of the skeletal muscular system, especially those of the masticatory apparatus and in particular movements of the tongue involved in swallowing (Anagnostara et al., 2001; Lear et al., 1965; Palmer et al., 2008; Pameijer et al., 1970), speaking (Lammert et al., 2011, 2013a, 2013b; Narayanan et al., 2004) and breathing (Bresolin et al., 1984; Cozza et al., 2007; Di Francesco et al., 2006; Emslie et al., 1952; Harari et al., 2010; Hartsook, 1946; Katz et al., 2004; Melink et al., 2010; Rubin, 1980; Valera et al., 2003; Vazquez‐Nava et al., 2006; Vig, 1998; Warren, 1990; Warren & Bishara, 2002) impact both upper and lower jaw morphology (Alghadir et al., 2015; Bansal et al., 2015; Ferrario et al., 1993; Hiiemae & Palmer, 2003; Hori et al., 2013; Klein, 1986; Oxilia et al., 2018; Palmer et al., 1997; Pirittiniemi, 1994) generating a best‐fit occlusion between jaws.

Para‐functional activity of the skeletal muscular system, instead, generates several anomalies which create an imbalance in the stomatognathic apparatus often producing occlusal interferences (Baldini, 2010; Carini et al., 2017; Cattoni et al., 2007; Cuccia & Caradonna, 2009; Gangloff et al., 2000; Hellsing et al., 1987; Milani et al., 2000; Nobili & Adversi, 1996; Perillo et al., 2011; Solow & Sonnesen, 1998; Valentino et al., 2002; Yoshino et al., 2003), temporomandibular (Bracco et al., 2004; Cuccia, 2011; Kritsineli & Shim, 1992; Lee et al., 1995; Olivo et al., 2006; Traversari et al., 2019) and musculoskeletal disorders (D'Attilio et al., 2005; Gadotti et al., 2005; Valentino & Melito, 1991).

However, to the evolutionary field, there is relatively little information on the relative impact of behavioral and cultural variables (such as dietary habits) on change in palate morphology during growth. Some scholars (Moreira et al., 2008; Schaefer et al., 2006) point out that palate asymmetry is equally distributed across age groups, even though it has been argued that fetuses and infants are more influenced by asymmetries than adults due to reasons that are currently unknown and independent from the appearance of dentition (Rossi et al., 2003). At present, distribution of variation in this anatomical district during growth are still far from being fully disentangled.

### 1.1 Deviations from bilateral symmetry.

Deviations from bilateral symmetry can be divided into three components: Directional asymmetry (DA), fluctuating asymmetry (FA), and antisymmetry (AS). DA refers to directionally consistent differences between the two sides of all individuals observed in a population and is generally associated with environmental factors and adaptive stress (Graham et al., 1994; Graham et al., 2010; Klingenberg et al., 2002). On the other hand, FA is defined as the distribution, at a group or population level, of the random individual deviations from the structure's symmetrical pattern (Klingenberg, 2015; Klingenberg & McIntyre, 1998; Mardia et al., 2000). FA is commonly interpreted as a measure of developmental instability displayed in human traits such as teeth, limb length and facial structure. Differences in diet and cultural behavior, as much as socioeconomic conditions, chromosomal anomalies, mutations, and reduced heterozygosity in a group can lead to higher rates of orofacial asymmetry during the development of the individual (Barden, 1980; Bogin, 1999; Hallgrímsson, 1988; Hershkovitz et al., 1993; Livshits et al., 1988; Lu et al., 2010; Noss et al., 1983; Özener, 2010; Sognnaes, 1978; Van Valen, 1962).

Finally, antisymmetry occurs when asymmetry shows no clear directionality across individuals and is generally associated with adaptive behaviors triggered by the interaction between genetic background and environmental conditions (Lu et al., 2010; Palmer, 1994; Van Valen, 1962). Since quantification of antisimmetry is still problematic and debated, there are no definitive methods available for multivariate shape data (Palmer et al., 2010; Palmer & Strobeck, 2003; Van Valen, 1962), and for this reason we have not included antisymmetry in the present analysis.

In this work, we investigate how asymmetry appear on the hard palate (the palatine processes of the maxilla, not including the horizontal plate of the palatines) at different stages of human growth and identify which regions of the palate are most affected by different kinds of functional and para‐functional alterations. More specifically, by using digital models of maxilla dental arches of two 19th and early 20th Century Italian populations (Florence and Bologna) we explore evidence of the ontogenetic development of directional and fluctuating asymmetry to understand whether palatal asymmetry significantly changes during growth. Understanding at least part of these alterations is critical to explain the adaptive processes of palate morphology during the entire lifespan of an individual.

## 2 MATERIALS

The sample consists of palatal arches of 183 individuals from two Italian identified human skeletal collections (Bologna and Florence) aged from 1 to 72 years. The individuals from the Bologna collection (*n* = 87), housed at the Museum of Anthropology of the University of Bologna, are from the Certosa cemetery located in the western suburbs of Bologna. The collection includes a total of 425 individuals of known sex, name and age at death (range 0–91 years), most of whom belonged to the less‐advantaged urban classes of late 19th and early 20th Century (Belcastro et al., 2017). The sample from the Florence collection (*n* = 96), hosted in the Natural History Museum (Anthropology and Ethnology section), University of Florence, belongs to unclaimed indigents from the Florence hospital and comprises lower‐class citizens of known sex, name and age at death (range 1–57 years), who lived in the town of Florence in the 19th century (industrialized only after 1890).

Paleopathological information of the Bologna individuals was sourced from archival data, which indicated that most died as a result of infectious disease (Belcastro et al., 2017). Based on health profiles, individuals from Florence collection breastfed children until 12–18 months of age and overall, adult experienced an unbalanced diet due to poor living conditions (Moggi‐Cecchi et al., 1994). Both these findings were consistent with pre‐ and post‐unification Italy in Bologna and pre‐industrialized Florence.

For the present study, male and female individuals from Bologna and Florence were separated into six groups (Table 1) based on direct observation of skeletal remains. Age groups were subdivided considering mixed dentition until permanent dentition based on the time of eruption/occlusion of the molars. The groups have been divided based on eruption of the dm2 (Group I; 1, 3–1.7 yo [years old]), permanent first molar (Group II; 2–6 yo), permanent second molar (Group III; 7–12 yo) and third molar (Groups IV; 13–18 yo) in occlusion (Groups V; 19–35 yo) and showing worn crown (Group VI; 36–72 yo). Individuals with damaged maxillae or presenting pathological conditions such as abscesses and extended alveolar bone absorption were excluded to avoid any spurious measurement of asymmetry.

**TABLE 1:** Six groups based on the time of eruption/occlusion of dm2, M1, M2, and M3 observing our sample

		Bologna			Florence			
		M	F	Tot	M	F	ND	Tot
Group I (1.3–1.7 year old)	dm2 erupt.	3	1	4	0	1		1
Group II (2–6 years old)	M1 erupt.	7	4	11	1	2		3
Group III (7–12 years old)	M2 erupt.	4	2	6	4	1		5
Group IV (13–18 years old)	M3 erupt.	5	6	11	6	8	1	15
Group V (19–35 years old)	M3 occ.	13	13	26	29	27		56
Group VI (36–72 years old)	Permanent complete dentition.	14	15	29	5	11		16

*Note*: Group I: deciduous dentition (dm2 eruption); Group II: mixed dentition (M1 eruption); Group III: mixed dentition (M2 eruption); Group IV: permanent dentition (M3 eruption); Group V: permanent dentition (M3 in occlusion), Group VI: Permanent complete dentition.

## 3 METHODS

### 3.1 Data acquisition

A total of 87 palatal arches from the Bologna collection were scanned at the Department of Biological, Geological and Environmental Sciences–BiGeA University of Bologna using a structured light 3D scanner (Artec Space Spider, Artec 3D, Luxembourg) with a high 3D resolution (up to 0.5 mm) and accuracy (up to 0.05 mm).

The Florence sample underwent CT scanning using Scanora 3D Cone Beam CT (SOREDEX, Tuusula, Finland) at the Museo di Storia Naturale, Antropologia e Etnologia, Florence, Italy. A total of 96 skull were scanned at 90 kVp, 0.2 mm Cu filtration, 225 half projection over 360°, 14 mAs radiation dose for a total scan time of 35 s per sample. Primary reconstruction of the images was performed using ART (Algebraic Reconstruction Technique). A total of 459 slices per scan were performed, with a 0.300 mm thickness, a 550 × 550px image size and a 0.300 mm pixel size.

Avizo 9.2 (Thermo Fisher Scientific) was used to generate isosurfaces. The 3D models of the skulls were refined in Geomagic Design X (3D Systems Software) to optimize the triangles and remove holes and defects while preserving the original surfaces.

### 3.2 Geometric morphometrics

Palatal surfaces were investigated through 3D landmark‐based geometric morphometric (GM) methods (Gunz et al., 2005; Mitteroecker & Gunz, 2009).

**FIGURE 1:**
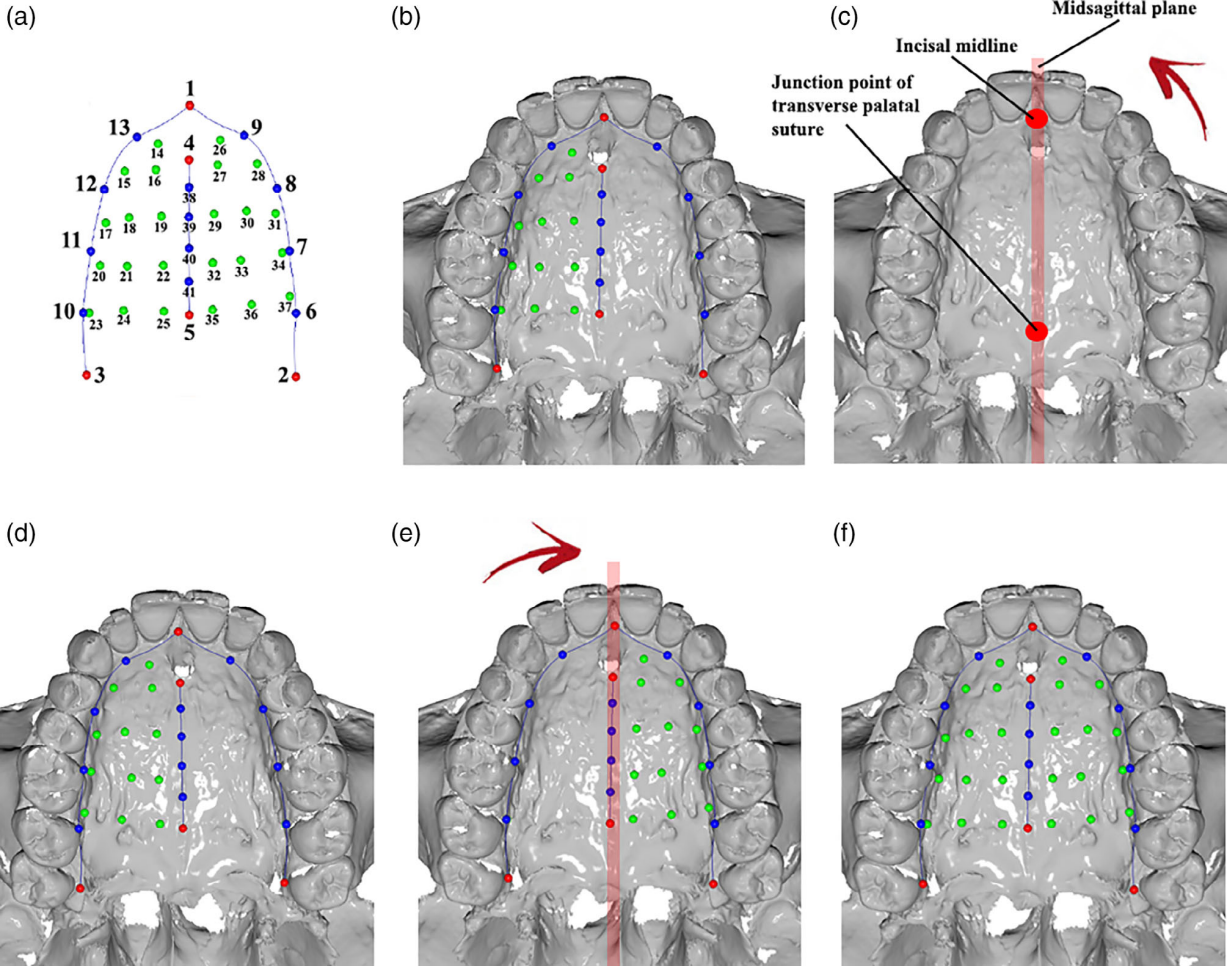
(a) the template with landmarks (red), curve semilandmarks (blue) and surface semilandmarks (green) digitized on the palatal arch. The procedure for the creation of the template are the following: (b) fixed landmark, curve semilandmarks and 12 surface semilandmarks on the right hemi‐palate were positioned; (c), the digital model of the palatal arch was mirrored according to the midsagittal‐plane; (d) then, the template was applied to the mirrored digital model; (e) the 12 surface semilandmarks were mirrored on the left hemi‐palate according to the midsagittal‐plane; (f) the 12 surface semilandmarks on the left hemi‐palate were added to the original configuration (b) to complete the left side

A 3D‐template (Figure 1(a)) of 41 (semi)landmarks (five landmarks, 12 curve semilandmarks and 24 surface semilandmarks) was created on the palatal surface of a young adult individual (20 yo) from the Bologna collection using Viewbox 4 software (dHal Software) and subsequently applied it to the entire sample (defined as targets).

The five landmarks were located between the two central incisors (named “Incisor,” number 1), on the lingual side of the last erupted molar (called “Ento‐left,” number 2 and “Ento‐right,” number 3), at the posterior end of the incisive foramen (called “Post‐foramen,” number 4), and, finally, at the junction of the median palatine and the transverse palatine sutures (called “Middle” number 5). Curves were digitized following the cervical lines on the right (curve right) and left (curve left) side of the palatal arch and curve semilandmarks were positioned 20% of the curve length with respect to one another. Finally, “Curve middle” was outlined by following the actual line of the median palatine suture. The 12 surface semilandmarks were positioned on the right hemi‐palate (Figure 1(b)) (Table 2). In order to obtain geometrically homologous surface semilandmarks in the right and left hemi‐palate (24 surface semilandmarks in total), the digital model was mirrored according to the midsagittal‐plane (passing through the incisal midline and the junction point of the transverse palatal suture) (Figure 1(c)) obtained as perpendicular to the occlusal reference plane following Oxilia et al. (2018).

**TABLE 2:** Landmarks and semilandmarks (sml = semilandmark) of the palatal shape configuration

Landmark no.	Name	Landmark no.	Name
1	Incisor	34	Surface sml left
2	Ento‐left	35	Surface sml left
3	Ento‐right	36	Surface sml left
4	Post‐foramen	37	Surface sml left
5	Middle	38	Curve sml middle
6	Curve sml left	39	Curve sml middle
7	Curve sml left	40	Curve sml middle
8	Curve sml left	41	Curve sml middle
9	Curve sml left		
10	Curve sml right		
11	Curve sml right		
12	Curve sml right		
13	Curve sml right		
14	Surface sml right		
15	Surface sml right		
16	Surface sml right		
17	Surface sml right		
18	Surface sml right		
19	Surface sml right		
20	Surface sml right		
21	Surface sml right		
22	Surface sml right		
23	Surface sml right		
24	Surface sml right		
25	Surface sml right		
26	Surface sml left		
27	Surface sml left		
28	Surface sml left		
29	Surface sml left		
30	Surface sml left		
31	Surface sml left		
32	Surface sml left		
33	Surface sml left		

*Note*: Compare with Figure 1.

The template configuration was applied to the mirrored model (Figure 1(d)), allowing the semilandmarks to slide on the curves (curve semilandmarks) and on the surface (surface semilandmarks) to minimize the thin‐plate spline (TPS) bending energy between the template and the target (i.e., the mirrored copy) (Slice, 2006). As a result, semilandmarks can be considered geometrically homologous (Gunz & Mitteroecker, 2013). The target configuration was mirrored back according to the above‐mentioned plane (Figure 1(e)), and the 12 surface semilandmarks on the left hemi‐palate were added to the original configuration to complete the template (Figure 1(f)). The final template configuration was applied to all 183 individuals.

### 3.3 Statistical analysis

#### 3.3.1 Geometric morphometric analysis

To analyze the asymmetry in the specific case of object symmetry, for each individual we generated a copy of the original landmark configuration by reflecting it on the opposite side of the x‐axis (i.e., multiplying values in the x column by −1) and relabelling it to obtain landmark correspondence (Klingenberg, 2015; Schaefer et al., 2006). Cartesian coordinates were converted into Procrustes shape coordinates by means of generalized procrustes analysis (GPA) using the function “gpagen” of the package “geomorph” (Adams & Otarola‐Castillo, 2013) in R version 3.6.2 (R Core Team, 2019). GPA removes information about translation by superimposing the centroid of each landmark configuration (centering). The latter is rotated to reduce to a minimum the sum of squared Euclidian distances between homologous landmarks, while scaling the configurations to centroid size equal to one (Mitteroecker & Gunz, 2009). A second step in the analysis allows the semilandmarks to slide against recursive updates of the Procrustes consensus (Gunz, Mitteroecker, & Bookstein, 2005; Mitteroecker & Gunz, 2009; Slice, 2006; Sorrentino, Stephens, et al., 2020; Sorrentino, Belcastro, et al., 2020).

We first computed a Procrustes distance matrix (Dryden & Mardia, 1998; Rohlf & Slice, 1990) between all the configurations obtained above, calculated the multivariate dispersion of each group and tested if group variances were homogeneous using the function betadisper in the “vegan” package in R (Oksanen et al., 2019). We then used Permutational Multivariate Analysis of Variance (PERMANOVA through the “adonis” function in the package “vegan” (Anderson, 2001) to assess whether the distribution of Procrustes distances was significantly different between the two geographical groups (Florence and Bologna) in each age group. Because of the absence of significant differences, the sample was put together, and subsequent analyses were conducted on this pooled sample to explore variability in asymmetry across age groups.

We then plotted, for each age group, a mean shape configuration obtained as the average of both originals and reflected/relabelled copies (which is defined as perfectly symmetric; Klingenberg et al., 2002) against the mean asymmetric component of shape variation (defined as the differences between the original and mirrored configuration; Klingenberg et al., 2002), in order to explore how the asymmetry pattern changes across landmarks.

### 3.4 Directional and fluctuating asymmetry

Following established methods (Klingenberg, 2015; Schaefer et al., 2006) we computed the mean of all individual palatal shape configurations in each age group ($\bar{O_{k}}$) and the mean of all the reflected and relabelled configurations ($\bar{{\mathrm{RR}}_{k}}$). We then calculated directional asymmetry for each age group (*DA*_*k*_) as the Procrustes distance between these two mean coordinate sets, that is, by first obtaining the squared difference between them ($d_{k}={\left(\bar{{\mathrm{RR}}_{k}}-\bar{O_{k}}\right)}^{2}$) and then computing the squared root of the grand sum of the resulting coordinate set (DA = sqrt(sum(((mean(RR) − mean(O))^2))) ${\mathrm{DA}}_{k}=\sqrt{\sum_{j=1}^{3}\sum_{l=1}^{41}d_{\mathrm{klj}}}$; Figure S1A). FA was calculated as the Procrustes distance of the difference between the original configuration and its reflected and relabelled copy of each individual (*d*_*i*_ = *O*_*i*_ − *RR*_*i*_) from their respective group DA (FA = sqrt(sum((([O‐RR] − DA)^2))) $\sqrt{\sum_{j=1}^{3}\sum_{l=1}^{41}{\left(d_{i}-{\mathrm{DA}}_{k}\right)}^{2}}$; Figure S1B).

To explore DA and FA within and across age groups in our pooled sample, we measured within‐group variability and between‐group differences for both asymmetries through Procrustes ANOVA (alpha = 0.05) using the “procD.lm” function in the package “geomorph.” According to the literature (Klingenberg et al., 2002; Palmer & Strobeck, 2003), Procrustes ANOVA attributes deviations from the overall mean configuration to main components (i.e., individual variability, reflection or DA, and interaction between individual variability and reflection or FA) (Klingenberg et al., 2002; Palmer & Strobeck, 2003). Finally, to further investigate the differences between all groups, therefore in order to evaluate for which specific group pairs these differences show significant values, we performed a post‐hoc Tukey's HSD test.

We then shifted to a finer scale of analysis to observe change over time in each (semi)landmark. Regarding DA, we assessed the presence of potential correlations in the distribution of values between pairs of age groups across all landmarks through Spearman rank correlation coefficient. We also detected the presence of significant differences in the distribution of DA values across landmarks through a Kruskal‐Wallis test, and performed a Dunn's post‐hoc test in order to identify which specific pairs of (semi)landmark these differences would show significant values.

We used Kruskal‐Wallis test also to assess the presence of significant between‐group differences in the distribution of FA values at each landmark and used Bonferroni correction to control for problems related to multiple testing.

Finally, a Mann–Whitney test was used to investigate the presence of significant differences in DA and FA between males and females for age groups IV, V, and VI, while sexual dimorphism was not evaluated for groups I‐III (age < 13) because of the absence/uncertainty of sexual traits.

## 4 RESULTS

Betadisper shows homogeneity of group variances (*p* value>0.05), and PERMANOVA results show no significant differences between the two examined groups, that is, Florence and Bologna (Table S1). This allowed us to consider the entire sample as a unique population and explore variability only across age groups in subsequent analyses.

Considering the whole asymmetric components of the morphological structure it was observed that Group I (1.3–1.7 yo) shows an absence of asymmetric bilateral direction between pairs of semi landmarks (Figure S2). In fact, black points corresponding to the right and left side presents a random direction of asymmetry. Opposite direction of asymmetry (anteriorly and posteriorly) between right and left side seems to be more visible from Group II (2–6 yo) with an increasing of vertical alteration in the middle and alveolar areas of the palate (black points are not visible or covering gray points) (Figure S2).

### 4.1 Directional asymmetry

Morphological differences within groups are statistically significant for groups II (2–6 yo) to VI (36–72 yo) (Table 3).

**TABLE 3:** Procrustes ANOVA (alpha = 0.05) of shape variation within groups

		*df*	SS	MS	Rsq	*F*	*p* value
Group I	Ind	4	0.022270	0.0055676	0.70211	3.1935	0.004
Group I	Reflections	1	0.002475	0.0024755	0.07804	1.4199	0.258
Group I	Ind X Reflections	4	0.006974	0.0017434	0.21985		
Group II	Ind	13	0.166690	0.0128223	0.82795	7.5942	0.002
Group II	Reflections	1	0.012689	0.0126890	0.06303	7.5152	0.002
Group II	Ind X Reflections	13	0.021950	0.0016884	0.10902		
Group III	Ind	10	0.190676	0.0190676	0.88842	11.3899	0.002
Group III	Reflections	1	0.007207	0.0072066	0.03358	4.3048	0.002
Group III	Ind X Reflections	10	0.016741	0.0016741	0.07800		
Group IV	Ind	25	0.43537	0.0174146	0.88387	10.1979	0.002
Group IV	Reflections	1	0.01451	0.0145093	0.02946	8.4965	0.002
Group IV	Ind X Reflections	25	0.04269	0.0017077	0.08667		
Group V	Ind	81	1.33530	0.016485	0.85730	7.2596	0.002
Group V	Reflections	1	0.03834	0.038335	0.02461	16.8817	0.002
Group V	Ind X Reflections	81	0.18394	0.002271	0.11809		
Group VI	Ind	44	0.90658	0.0206040	0.88354	9.5034	0.002
Group VI	Reflections	1	0.02410	0.0240969	0.02348	11.1145	0.002
Group VI	Ind X Reflections	44	0.09539	0.0021681	0.09297		

*Note*: Reflections, directional asymmetry; IndXReflections, fluctuating asymmetry.

Abbreviations: *df*, degrees of Freedom; *F*, Fisher value; Ind, individual variability; MS, mean square; Rsq, *r* squared; SS, sums of squares.

**FIGURE 2:**
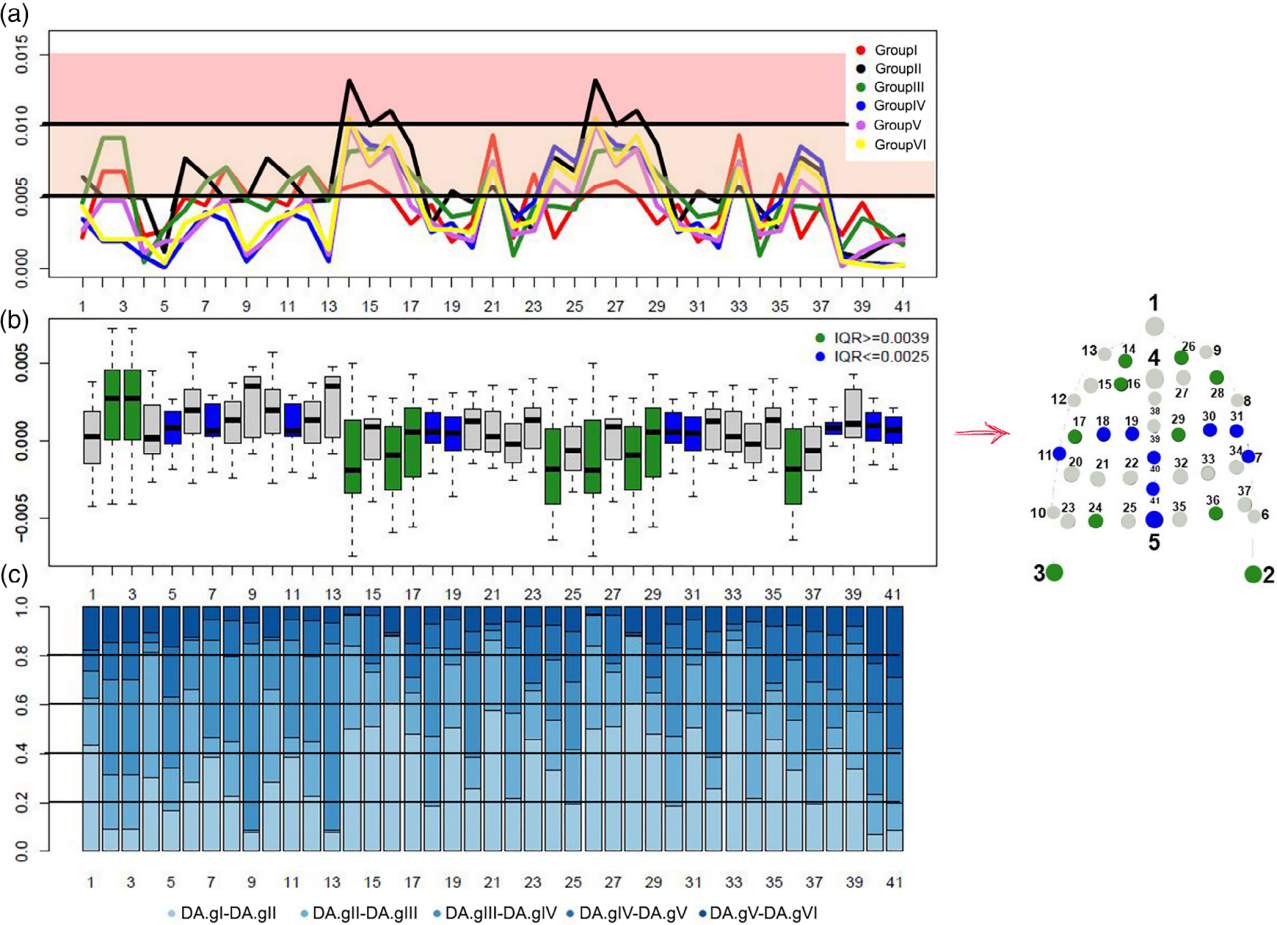
Variability of directional asymmetry in different age groups. (a) Line plot depicting the distribution of DA values across (semi)landmarks for each individual age group (corresponding colors are detailed in the legend on the right side of the panel); (b) boxplot showing the distribution of pairwise differences in DA values between age groups measured at each (semi)landmark. Dispersion of difference values was measured through inter‐quartile ranges (IQR), that is the difference between the first and third quartiles at each sampling point. Green refers to particularly dispersed patterns (IQR ≥ 0.0039) while blue indicates patterns well described by median values (IQR ≤ 0.0025); (c) stacked bar plot showing the relative weight of differences in DA values between subsequent groups of dentition (from deciduous to permanent) for each (semi)landmark value

There are also statistically significant differences between groups (*p* = 0.0158; Table 4), where Turkey's HSD post‐hoc test identifies different patterns between group V (19–35 yo) and II (Table 5). Spearman's rank correlation coefficients show that DA values are correlated (>0.80) between the following pairs of age groups: V – IV, VI‐II, VI‐IV, and VI‐V (Table 6). As shown in Figures 2(a) (see also Figure S3), the anterior area of the palate (i.e., semilandmarks 14, 15, 16, 26, 27, 28) presents the highest values of DA, mainly in groups II and VI. Moderate DA values involve the posterior palatal surface (i.e., landmarks 2, 3), especially in groups I (1,3–1,7 yo) and III (7–12 yo). As far as groups IV and V are concerned, lower levels of DA have been identified in the anterior portion of the palate.

**TABLE 4:** Procrustes ANOVA (alpha = 0.05) of DA variation between groups

	*df*	SS	MS	*F* value	*p* value
DA.group	5	0.000105	0.000021	2.86	0.0158
Residuals	240	0.001762	0.000007344		

Abbreviations: df, degree of Freedom; *F*, Fisher value; MS, mean square; SS, sum of square.

**TABLE 5:** Directional asymmetry

	diff	lwr	upr	*p* adj
gII‐gI	0.001418518	−0.0003009776	0.003138013	0.1710723
gIII‐gI	0.0006442728	−0.0010752223	0.002363768	0.8903698
gIV‐gI	−0.0002701058	−0.0019896010	0.001449389	0.9976262
gV‐gI	−0.000449493	−0.0021689882	0.001270002	0.9751570
gVI‐gI	−0.0002452147	−0.0019647098	0.001474281	0.9985065
gIII‐gII	−0.0007742447	−0.0024937399	0.0009452505	0.7883765
gIV‐gII	−0.001688623	−0.0034081185	0.00003087185	0.0575082
gV‐gII	−0.001868011	−0.0035875057	−0.0001485154	**0.0244739**
gVI‐gII	−0.001663732	−0.0033832274	0.00005576298	0.0642329
gIV‐gIII	−0.0009143786	−0.0026338738	0.0008051165	0.6467330
gV‐gIII	−0.001093766	−0.0028132611	0.0006257293	0.4501011
gVI‐gIII	−0.0008894875	−0.0026089827	0.0008300077	0.6734831
gV‐gIV	−0.0001793873	−0.0018988824	0.001540108	0.9996731
gVI‐gIV	0.00002489113	−0.0016946040	0.001744386	1.0000000
gVI‐gV	0.0002042784	−0.0015152168	0.001923774	0.9993833

*Note*: Results of the Tukey's HSD post‐hoc test performed to evaluate which group pairs show statistically significant differences in the distribution of DA values between groups. All the *p* values are Bonferroni corrected. Significant differences shown in bold.

Abbreviations: diff, difference in the observed means; g, group; lwr = lower end point of the interval; *p* adj = Bonferroni corrected *p* value; upr, upper end point of the interval.

**TABLE 6:** Results of the spearman rank correlation coefficient performed on the distribution of DA values across landmarks between pairs of age groups

	Group I	Group II	Group III	Group IV	Group V
Group II	0.20				
Group III	0.71	0.60			
Group IV	0.28	0.77	0.55		
Group V	0.46	0.71	0.76	0.88	
Group VI	0.36	0.81	0.61	0.96	0.89

*Note*: Correlation coefficient comprised between −1 (strong negative correlation) and 1 (strong positive correlation), where 0 means lack of correlation.

More detailed results are highlighted in Figure 2(b), where boxes are colored based on the range of dispersion of each (semi)landmark. Anterior and posterior areas of the palatal arch show more apparent dispersion in the distribution of differences between groups (IQR > =0.0039) compared to the posterior area of the middle palatine suture and the area localized on the palatine process (blue boxes), where we measured lower interquartile dispersion (IQR ≤ 0.0025). Moreover, the area corresponding to the left and right sides of the palatal arch (semilandmarks 14–25 and 26–37, Figure 2(b),(c)) shows a symmetrical alteration during growth as demonstrated by the same bilateral variation, which appears prevalent on the alveolar arches during the transition from mixed to permanent dentition (semilandmarks 6–9 and 10–13, Figure 2(c)). All these results are confirmed by the Kruskal‐Wallis test which shows a significant difference in the distribution of DA values across landmarks (*p* value<0.0001). In particular Dunn's post‐hoc test (Table 7) indicates a significant difference between semilandmarks located in the left and right sides of the anterior and posterior area of the palatal arch (semilandmarks 14–16, 26–28, and 21, 33) and (semi)landmarks located in the middle palatine suture (semilandmarks 38–41 and landmarks 4–5), highlighting lesser values of asymmetry in the latter than it has been observed in the anterior and posterior area.

**TABLE 7:** Directional asymmetry

Comparison	*Z*	*p*. unadj	*p*. adj
14–38	4.880435	1.058522e‐06	0.0008679876
15–38	4.515315	6.322269e‐06	0.0051842608
16–38	4.661363	3.141221e‐06	0.0025758013
21–38	4.288129	1.801841e‐05	0.0147750965
26–38	4.880435	1.058522e‐06	0.0008679876
27–38	4.515315	6.322269e‐06	0.0051842608
28–38	4.661363	3.141221e‐06	0.0025758013
33–38	4.288129	1.801841e‐05	0.0147750965
14–39	4.280016	1.868802e‐05	0.0153241743
16–39	4.060944	4.887475e‐05	0.0400772916
26–39	4.280016	1.868802e‐05	0.0153241743
28–39	4.060944	4.887475e‐05	0.0400772916
14–4	4.247561	2.161108e‐05	0.0177210829
16–4	4.028489	5.613657e‐05	0.0460319856
26–4	4.247561	2.161108e‐05	0.0177210829
28–4	4.028489	5.613657e‐05	0.0460319856
14–40	4.628908	3.675992e‐06	0.0030143132
15–40	4.263788	2.009901e‐05	0.0164811850
16–40	4.409836	1.034489e‐05	0.0084828115
21–40	4.036602	5.423085e‐05	0.0444692996
26–40	4.628908	3.675992e‐06	0.0030143132
27–40	4.263788	2.009901e‐05	0.0164811850
28–40	4.409836	1.034489e‐05	0.0084828115
33–40	4.036602	5.423085e‐05	0.0444692996
14–41	4.738444	2.153657e‐06	0.0017659985
15–41	4.373324	1.223689e‐05	0.0100342480
16–41	4.519372	6.202334e‐06	0.0050859139
21–41	4.146138	3.381295e‐05	0.0277266207
26–41	4.738444	2.153657e‐06	0.0017659985
27–41	4.373324	1.223689e‐05	0.0100342480
28–41	4.519372	6.202334e‐06	0.0050859139
33–41	4.146138	3.381295e‐05	0.0277266207
14–5	4.551827	5.318204e‐06	0.0043609275
15–5	4.186707	2.830305e‐05	0.0232085009
16–5	4.332755	1.472548e‐05	0.0120748963
26–5	4.551827	5.318204e‐06	0.0043609275
27–5	4.186707	2.830305e‐05	0.0232085009
28–5	4.332755	1.472548e‐05	0.0120748963

*Note*: Results of the Dunn's post‐hoc test performed to evaluate which specific pairs of (semi)landmark show statistically significant differences in the distribution of DA values across landmarks. Here reported just the pairs of (semi)landmark where the *p* value indicates a statistical significant difference. All the *p* values are Bonferroni corrected.

Abbreviations: Comparison, pairwise (semi)landmarks comparison; *p*. adj, Bonferroni corrected *p* value for each comparison; *p*. unadj, unadjusted *p* values for each comparison; *Z*, values for the *Z* test statistic for each comparison.

**FIGURE 3:**
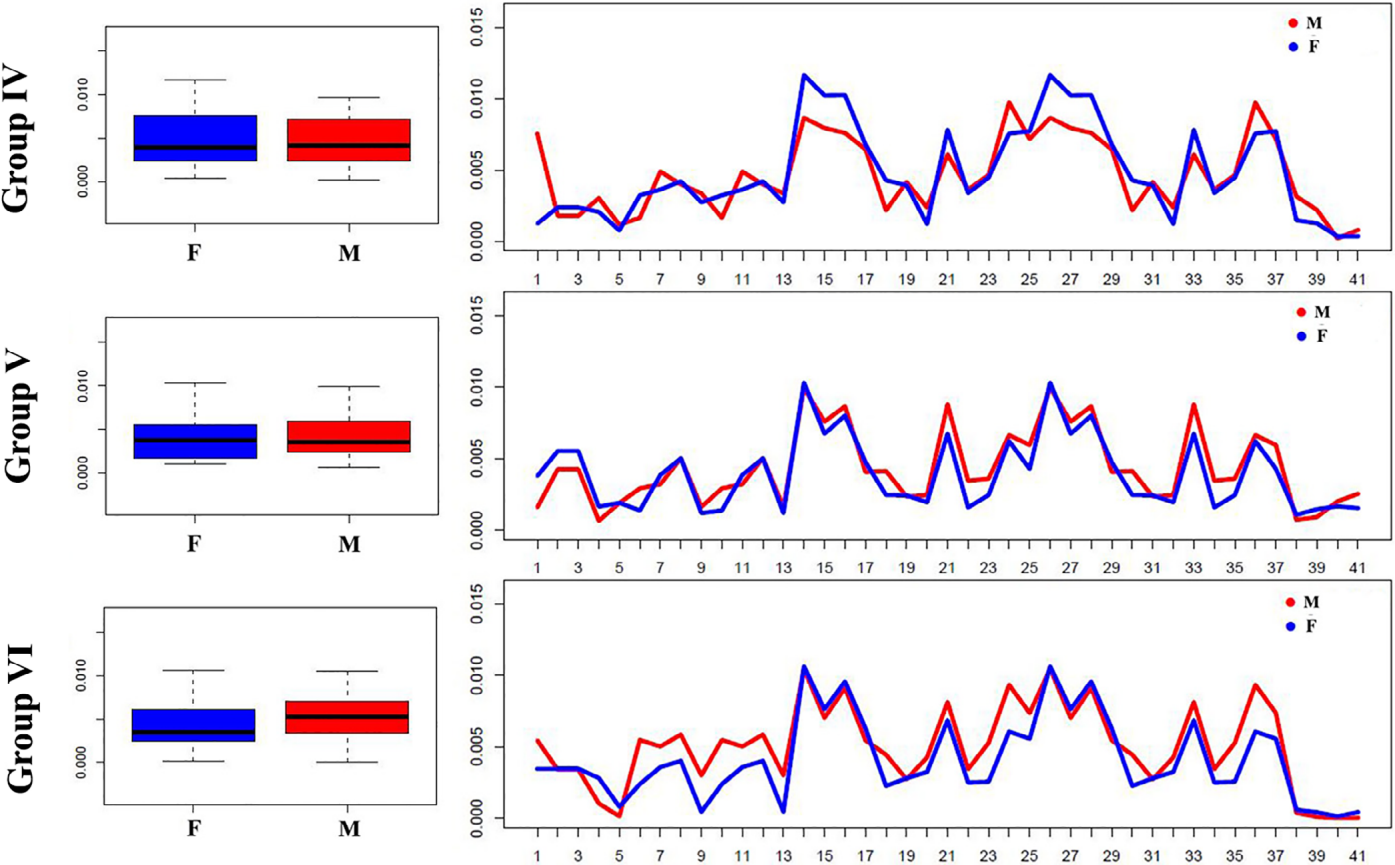
Differences in the distribution of DA values between males and females in different age groups. Left: Boxplots showing the overall distribution of DA values across (semi)landmarks for different age classes; right: Line plots showing differences in the distribution of DA values between males and females at each (semi)landmark. M = males, red colored boxes (left) and lines (right); F = females, blue colored boxes (left) and lines (right)

As shown in Figure 3, males and females (groups IV‐V‐VI) show similar distribution of DA values. This is also confirmed by the absence of significant differences in Mann–Whitney‐Wilcoxon test (*p* value >0.05).

### 4.2 Fluctuating asymmetry

As far as within group differences are concerned (Table 3), the portion of variance explained by FA ranges from 7% to 9% for groups III‐IV‐VI, increasing to around 11% for group V and 10% for group II, with a higher percentage for group I at around 21%.

**FIGURE 4:**
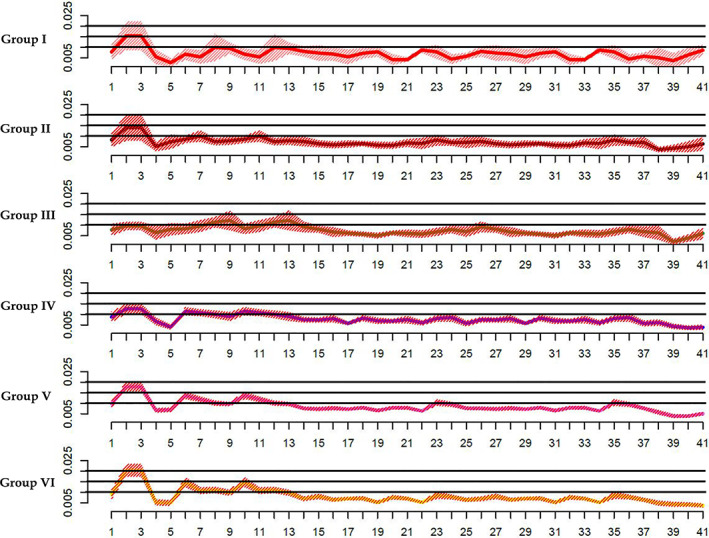
The distribution of fluctuating asymmetry in different age groups. Solid lines show mean FA values calculated at each (semi)landmark for each age group, while the shaded envelope represents a 95% confidence interval

**FIGURE 5:**
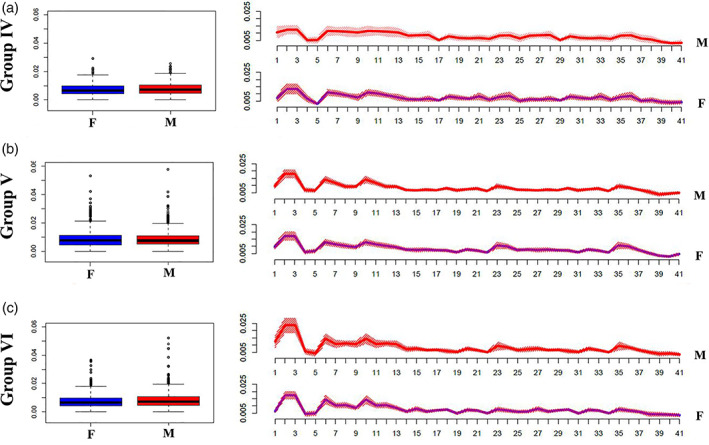
Differences in the distribution of FA values between males and females in different age groups. Left: Boxplots showing the overall distribution of FA values across (semi)landmarks for different age classes; right: Line plots showing differences in the distribution of FA values between males and females at each (semi)landmark. M = males, red colored boxes (left) and lines (right); F = females, blue colored boxes (left) and lines (right)

Moreover, Table 8 shows statistically significant differences between groups (*p* value <0.0001) especially concerning groups V‐I, VI‐I, V‐II, VI‐II, V‐III, V‐IV after Tukey's HSD post‐hoc test (Table 9). Kruskal‐Wallis test shows that significant differences in the distribution of FA values emerged only for landmark 2, 3 and 6, 10 corresponding to ento‐molars left/right and alveolar molar area respectively (Table S2). Peaks observed in correspondence of landmark 2 and 3 decrease their effect up to group III and gradually increases again until group VI, while peaks 6 and 10 (peaks localized on alveolar areas) only increase from group IV (Figure 4). Regarding inter‐individual variance the evidence of its dispersion of individuals around the mean was graphically confirmed within group I up to group III (Figure 4) decreasing then in the other groups. As shown in Figure 5, males and females (group IV‐V‐VI) show similar distribution of FA values. This is also confirmed by the absence of significant differences in Mann–Whitney‐Wilcoxon test (*p* value >0.05).

**TABLE 8:** Procrustes ANOVA (alpha = 0.05) of FA variation between groups

	*df*	SS	MS	F value	*p* value
FA.group	5	0.00207	0.0004137	13.6	0.00000000000031
Residuals	7497	0.22810	0.0000304		

Abbreviations: *df*, degrees of Freedom; *F*, Fisher value; MS, mean square; SS, sums of squares.

**TABLE 9:** Fluctuating asymmetry

	diff	lwr	upr	*p* adj
gII‐gI	0.0001977353	−0.001081561	0.001477032	0.9979107
gIII‐gI	0.0004704010	−0.000854008	0.00179481	0.9139878
gIV‐gI	0.0006716972	−0.0005273952	0.00187079	0.6007386
gV‐gI	0.0016449604	0.0005138338	0.002776087	**0.0004909**
gVI‐gI	0.0011973402	0.00003979695	0.002354883	**0.0377149**
gIII‐gII	0.0002726658	−0.0007166915	0.001262023	0.9700381
gIV‐gII	0.0004739620	−0.000340035	0.001287959	0.5586858
gV‐gII	0.0014472251	0.0007371436	0.002157307	**0.0000001**
gVI‐gII	0.0009996049	0.0002481564	0.001751053	**0.0020882**
gIV‐gIII	0.0002012962	−0.0006819084	0.001084501	0.9871338
gV‐gIII	0.0011745593	0.0003860956	0.001963023	**0.0003170**
gVI‐gIII	0.0007269392	−0.00009897496	0.001552853	0.1215326
gV‐gIV	0.0009732631	0.0004205984	0.001525928	**0.0000079**
gVI‐gIV	0.0005256430	−0.00007925166	0.001130538	0.1310185
gVI‐gV	−0.0004476201	−0.0009031661	0.000007925781	0.0574075

*Note*: Results of the Tukey's HSD post‐hoc test performed to evaluate which group pairs show statistically significant differences in the distribution of FA values between groups. All the *p* values are Bonferroni corrected. Significant differences shown in bold.

Abbreviations: diff, difference in the observed means; g, group; lwr, lower end point of the interval; *p* adj, Bonferroni corrected *p* value; upr, upper end point of the interval.

## 5 DISCUSSION

By observing morphological variation in the palate, we achieved an in‐depth understanding of asymmetry and where it manifests. Slight morphological variation (orange points of Figure S3) is visible between right and left sides at the posterior area of the palate. The results described in this study provide evidence of inter‐individual morphological differences during palate growth. In the main, asymmetry appears as a functional response to different inputs such as physiological forces, nutritive and non‐nutritive habits, para‐masticatory activity, and speech development. In this study we highlight this response during growth.

Whether this effect depends on particular features of the present sample or on sample size will need to be further tested in the future with larger samples.

Asymmetric components show a multidirectional movement which involved the entire morphological structure. In detail, in our sample the effects of asymmetry produce a multidirectional alteration of the palate morphology in the Group I (1.3–1.7 yo) and become oriented (opposite bilateral direction) anteriorly (left side) and posteriorly (right side) from Group II (2–6 years age). In order to define in which way asymmetry (directional and fluctuating) behave during life we provided a more detailed description below.

### 5.1 Directional asymmetry

Group I (1.3 and 1.7 yo) shows slight morphological variation on the palatal surface opposed to group II which shows higher values of alteration particularly in the incisal zone (Figure 2(a), and red points in Figure S3). Similar to group I, group III (7–12 yo) shows a moderate alteration (orange points in Figure S3) on the palatal surface mainly localized on the anterior and posterior area. Morphological changes seem to reach a plateau with M3s eruption (group IV) and its occlusion contact (group V). Eventually, a slight modification localized on the anterior area seems to characterize group VI (senior).

Individuals with incisor proclination, dental crowding, and a narrow and deep palate resulting in irreversible asymmetry, likely generated by insufficient naso‐respiratory function and hypertrophied adenoids (Bresolin et al., 1984; Cozza et al., 2007; Di Francesco et al., 2006; Emslie et al., 1952; Figus et al., 2017; Harari et al., 2010; Hartsook, 1946; Katz et al., 2004; Melink et al., 2010; Rubin, 1980; Valera et al., 2003; Vazquez‐Nava et al., 2006; Vig, 1998; Warren, 1990; Warren & Bishara, 2002) were excluded from the sample. These pathological conditions have a measurable impact on palate morphology before the age of 12 and after the age of 5 yo. Asymmetries identified in individuals belonging to class I and II were interpreted as produced by no‐pathological variables because the area of morphological variation combined with lower intensity of asymmetry does not seem to be linked to a para‐functional factor.

One of the most likely explanations for the observed alteration (Figure S3) identified on the palatal surface of group I could be linked to remnants of intrauterine development (Wolpert et al., 2006). As a matter of fact, fetal life consolidates multifactorial patterns of growth and leads to asymmetry (Moreira et al., 2008). Considering the absence of biomechanical stress typically produced by dental occlusion, we might attribute asymmetry of these individuals to the physiological development of primary teeth. The latter start to form in the uterus, more specifically at the end of the fifth week of gestation (Koussoulakou et al., 2009). By the time, the embryo is 10 weeks old, there are 10 buds on the upper and lower arches that will eventually become the primary (deciduous) dentition. These teeth will continue to form until they erupt in the mouth within the first and second years of life.

Lateral pressure of natural birthing (Katz et al., 2004) might be another factor. The pressure experienced during birth causes asymmetry in the anterior and posterior area of the palate (De Souza et al., 1976; Lissauer & Hansen, 2020).

The “sucking movements during breastfeeding” is another variable which generates unbalanced pressure of the tongue on each half of the palate. However, our results show symmetric alteration (Figure 2) on the anterior region of the palate (Figure S3). Morphological differences identified between infants (lowering and expanding of the palate structure) and adults are rather to be attributed to the passage of air through the nose during breastfeeding which exerts pressure on the palate (Walker, 2014).

Considering the intensity of anterior asymmetry identified in group II (Figure 2 and Figure S3), it might be ascribed to habit/skills usually acquired between 2 and 6 yo such as non‐nutritive sucking, development of speech and/or dietary habits. These variables, indeed, can produce an anterior asymmetrical or unbalanced over‐exertion generated by thumb (i.e., non‐nutritive sucking) or tongue pressure (i.e., languages, tongue thrust, dietary habits, breathing pattern).

Non‐nutritive sucking habits may result in the development of physiological imbalance during growth in addition to deviations from a normal swallowing pattern (Cozza et al., 2007; Katz et al., 2004; Melink et al., 2010; Vazquez‐Nava et al., 2006; Warren & Bishara, 2002) due to thumb‐sucking pressure (Yokota et al., 2007) and tongue anterior thrusting leading to longer‐term problems especially present in children (Melink et al., 2010; Vazquez‐Nava et al., 2006).

As far as development of speech is concerned, it is known that between 3 and 6 years of age, speech sounds and words are pronounced clearly (Berk, 2013; Bloom & Lahey, 1978). Tongue strength and its influence on palate morphology increases rapidly from 3 to 6.5 years of age, after which it decreases and has less of an impact for the remainder of the life span (Potter et al., 2019). As our sample include native Italian individuals, Italian language might be considered as another plausible source of the observed variation, since articulatory mechanisms for the production of voiced stopped consonants (/t/ and /d/), produce a higher anterior pressure of oral air with an anterior contact between tongue and palate (Moen et al., 2001). Eventually, the maximum tongue strength (45%–60% of power) during swallowing, dependent on bolus size and viscosity (Youmans et al., 2009), generates a distributed pressure on the palatal surface which in our case appears identified by orange circles on the palatal surface (Figure S3). In light of these considerations, the anterior alteration shared by group II until group VI might be ascribed to these variables (Figure 2 and Figure S3).

The anterior and posterior variation of the maxilla bone identified in group III (7–12 yo) might be influenced by dental turnover. As far as this variable is concerned, dental eruption and/or premature loss of primary dentition produce a slight morphological adjustment (Profit et al., 1986) (Figure 2 and Figure S3).

The signal of M3 eruption and occlusion seems to be detected in group IV and V respectively, where the force thrust of this tooth slightly produce an anterior alteration (Gavazzi et al., 2014; Niedzielska, 2005) (see Figure S3). It is interesting to observe how M3 eruption (group IV) and its occlusion (group V) does not alter the masticatory system as a whole to such an extent as to produce high asymmetry on the anterior area, as previously described by Niedzielska (2005) and Gavazzi et al. (2014).

Finally, asymmetry was identified on the anterior area of the palate for individuals in group VI. This could be ascribable to the plasticity of the facial skeleton which changes during adult age (more than 50 yo) as suggested by bone resorption and bone growth surrounding the canine fossa (Schuh et al., 2019) as well as shortening and narrowing/flattening of the face in senility (Hellman, 1927).

As far as differences between males and females are concerned, group IV exhibits the major number of alterations (around 13–18 yo): females show morphological asymmetry especially on both sides of the anterior area of palatal arch (semilandmarks 14–18 and 26–30); a change not acquired in males until later in life (group V and VI). As suggested by Bastir et al. (2006), this result is probably lead by shape maturation of female facial and mandibular structures (around 15–16 yo). In fact, sexual dimorphism in the face as well as bidirectional developmental influences between the lateral cranial floor and the face happens until about 11–12 years and is related to the slightly prolonged growth and development of males compared with females (Bhatia & Leighton, 1993; Enlow & Hans, 1996; Rosas & Bastir, 2002).

### 5.2 Fluctuating asymmetry

FA shows differences between groups but seems to fade out up to group IV, where morphological instability of palatal arch reduces (group V and VI) (Figure 4). Indeed, premolar (semilandmarks 8 and 12), molar (Landmarks 2 and 3) and palatine mid‐sagittal suture (landmarks 38, 39 and 40) show high variability around mean values of FA during the first year of life (Figure 4) probably due to the ductility of maxillary bone. Our results confirm that during growth, asymmetry of the posterior palatal area of infants reduced up to group III where only anterior area (landmarks 9 and 13) shows a high degree of asymmetry. This signal might be ascribed to the disto‐mesial closing of palatal suture which starts posteriorly in young age and ends in the anterior area during older ages (Melsen, 1975; Melsen & Melsen, 1982; Persson & Thilander, 1977). Therefore, morphological variability decreases during growth, when it is hidden by mechanical stress (probably linked to physiological process and/or paramasticatory habits) identified by directional asymmetry.

As far as differences between males and females are concerned, group IV (13–18 yo) and group VI (36–72 yo) males seem to have a higher dispersion around the mean than females, which could be interpreted as evidence of a greater morphological ductility of the palatal arch. This is quite evident especially for landmarks 2–3 (Figure 5) of group VI.

Results of this study underline the dominance of FA in the earlier stages of life and the role of DA as mechanical stress accumulated during growth.

## 6 CONCLUSION

Given the evidence of mechanical alteration on morphology of the palate, we suggest that the multidirectional asymmetry (lateral, anterior, posterior, and vertical asymmetry, see Figure S2) in the masticatory system is the product of a complex chain of responses.

Our results highlight over the lifespan that the effects of FA diminish and DA becomes more noticeable. The stability of alveolar bone and palatal morphology during life seems to change because of non‐nutritive sucking, development of languages and food consistency, all of which concur to bone modification depending on age stages. Physiological processes (e.g., breastfeeding sucking, dental turnover, M3 eruption/occlusion), on the other hand, have a slight but measurable influence on morphology.

This work resonates with research aimed at understanding possible relationship between age and language acquisition based on tongue pressure and palate morphology. It provides new perspectives from a medical and evolutionary point of view. In a medical perspective, it could be considered as a useful guide for planning orthodontic and surgical procedures, especially in light of the need in maintaining stability of asymmetric corrections. Predicting the possible changes throughout the lifespan, can be useful for applying the appropriate device for each age (e.g., removable retainer, fixed orthodontic appliances). The results should also be considered when applying less‐invasive approaches such as myofunctional cure or speech therapist to be matched to the most affective phase and age. In evolutionary perspective a reduction of biomechanical loadings and forces involving our masticatory system during food ingestion, chewing and non‐masticatory dental activities could be considered one of many variables leading to asymmetry. Continuing biomechanical pressure on our masticatory system triggers variation and asymmetry in development, growth, and remodeling activity in the bony structures. It is likely that each recent and/or ancient human group shows a specific pattern of asymmetry that could be useful to obtain information on cultural habits, physiological processes, as well as language development along time.

Future studies should consider the amount of asymmetry in the masticatory system of modern humans to distinguish physiological (integral part of individuals development) and pathological conditions (interference with the normal dental function and esthetic appearance) as well as understanding any non‐facial effects associated with changes to the palate such as cranial base, expansion of neurocranium and/or suture closure timing (Libby et al. 2017, Bastir et al. 2004) by using a comprehensive “template” able to detect any morphological dependence among cranial bones.

Moreover, investigating the effects of occlusion on the upper and lower jaws in addition to cranial asymmetries in non‐human primates, will be useful to acquire a broader perspective on how individual asymmetry changes during growth in different species. This will augment the new knowledge on the interaction between these processes and the development of different socio/environmental contexts and will provide considerable insight on yet to be understood evolutionary processes.

## CONFLICT OF INTEREST

The authors declare no competing interests.

## AUTHOR CONTRIBUTIONS

Gregorio Oxilia, Sergio Martini, Jessica C. Menghi Sartorio, Eugenio Bortolini, Stefano Benazzi conception and design; Stefano Benazzi funded the project; Gregorio Oxilia, Melchiore Giganti, Tommaso Mori, Giulia Zampirolo, Jacopo Moggi Cecchi acquisition of data. Gregorio Oxilia, Giulia Zampirolo, Jessica C. Menghi Sartorio reconstructed the digital model; Jessica C. Menghi Sartorio, Eugenio Bortolini and Gregorio Oxilia performed statistical analysis; Gregorio Oxilia, Jessica C. Menghi Sartorio, Eugenio Bortolini, Andrea Papini, Marco Boggioni, Sergio Martini, Filippo Marciani, Stefano Benazzi analyzed the data; Gregorio Oxilia, Jessica C. Menghi Sartorio, Eugenio Bortolini, Andrea Papini, Marco Boggioni, Sergio Martini, Filippo Marciani, Stefano Benazzi discussed the results; Gregorio Oxilia, Jessica C. Menghi Sartorio, Eugenio Bortolini and Stefano Benazzi wrote the manuscript Gregorio Oxilia, Jessica C. Menghi Sartorio, Eugenio Bortolini, Simona Arrighi, Carla Figus, Giulia Marciani, Sara Silvestrini, Maria Elena Pedrosi, Alessandro Riga, Ottmar Kullmer, Rachel Sarig, Luca Fiorenza, Rita Sorrentino, Maria Giovanna Belcastro, Jacopo Moggi Cecchi, Stefano Benazzi edited the manuscript. All authors read, revised and approved the final version of the manuscript. Maria Giovanna Belcastro and Jacopo Moggi Cecchi are scientific directors of the collections of Bologna and Florence respectively.

## Supporting information

**Data S1.** Supporting Information.

## Data Availability

The data that support the findings of this study are openly available in AMSActa ‐ Institutional Research Repository at DOI 10.6092/unibo/amsacta/6629, reference number 6629.
