# Supplementary material for: Exploring directional and fluctuating asymmetry in the human palate during growth
Source: Am J Phys Anthropol. 2021 May 11;175(4):847–64. doi: 10.1002/ajpa.24293 (PMC8360102; doi:10.1002/ajpa.24293)
Supplement: Supplementary file 1 — Data S1. Supporting Information. [file AJPA-175-847-s001.docx]

**Supplementary Information**

Gregorio Oxilia, Jessica C. Menghi Sartorio, Eugenio Bortolini, Giulia Zampirolo, Andrea Papini, Marco Boggioni, Sergio Martini, Filippo Marciani, Simona Arrighi, Carla Figus, Giulia Marciani, Matteo Romandini, Sara Silvestrini, Maria Elena Pedrosi, Tommaso Mori, Alessandro Riga, Ottmar Kullmer, Rachel Sarig, Luca Fiorenza, Melchiore Giganti, Rita Sorrentino, Maria Giovanna Belcastro, Jacopo Moggi Cecchi, Stefano Benazzi


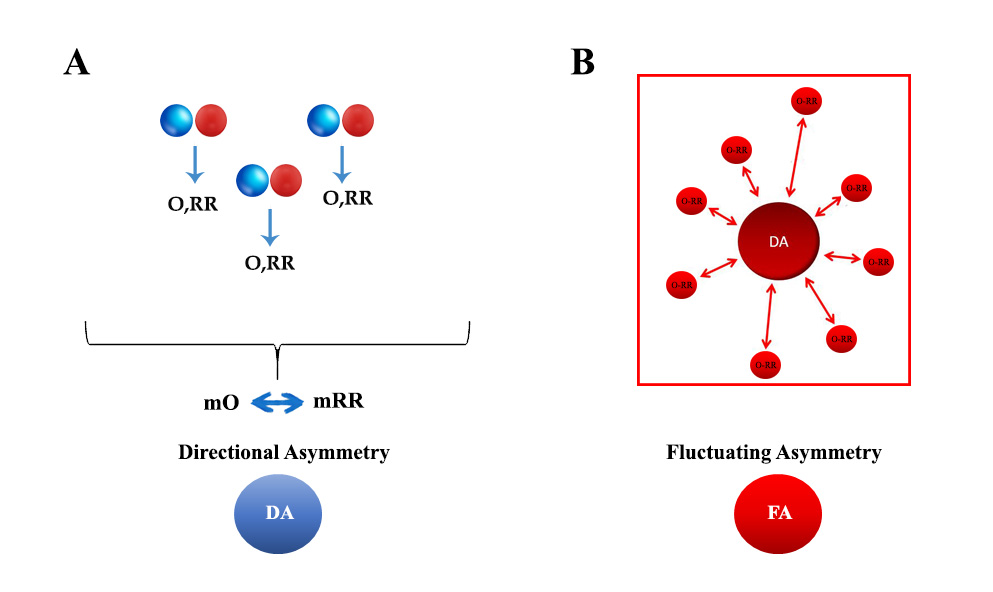


**Figure S1**. **A)** Directional Asymmetry (DA) obtained as the Procrustes distance between the mean of all the original palatal shape configurations (mO) and the mean of all the reflected and relabelled configurations (mRR). **B)** Fluctuating asymmetry (FA) obtained as the Procrustes distance of the difference between the original configuration and its reflected and relabelled copy (O-RR) from DA.


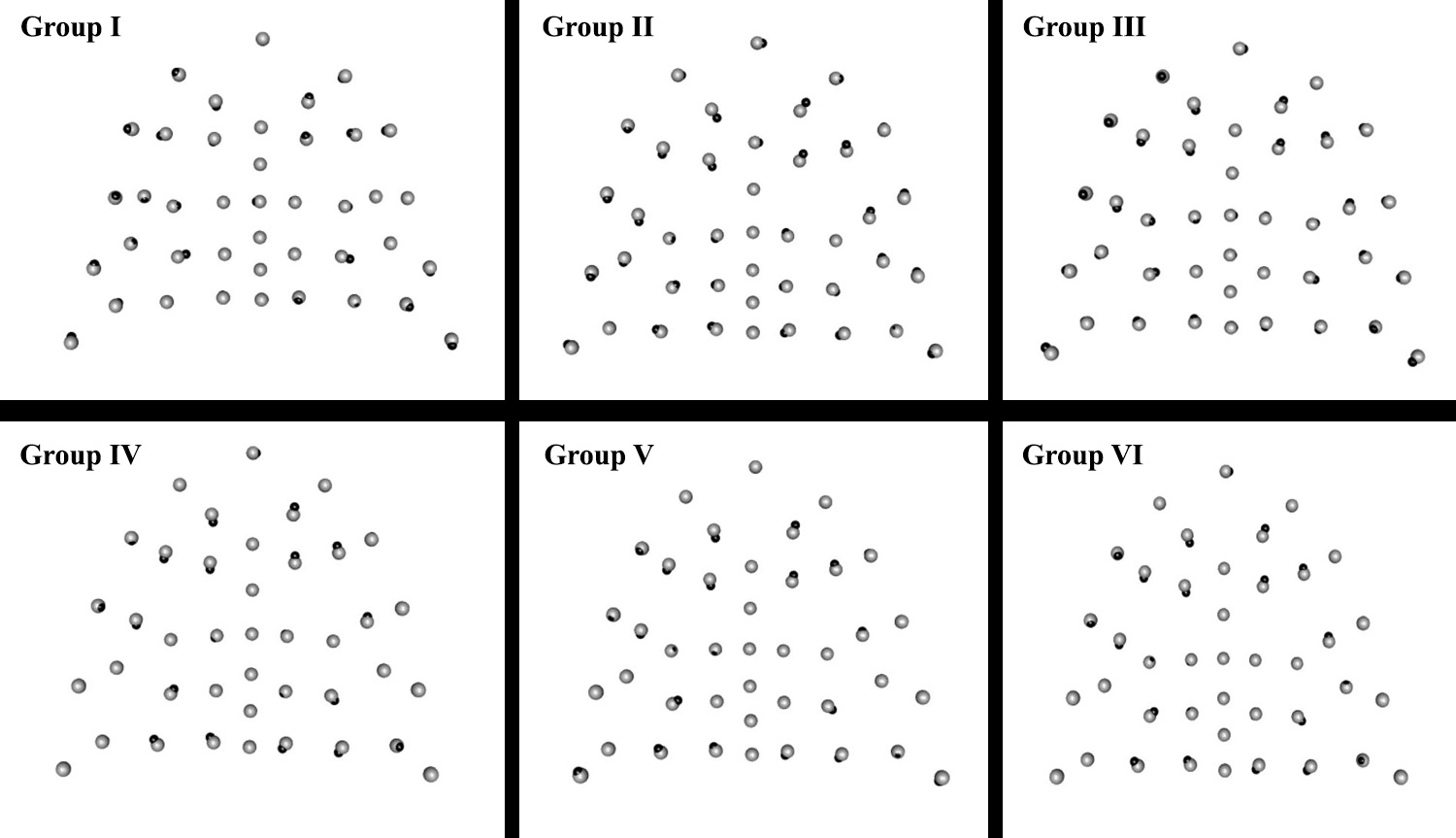


**Figure S2.** Asymmetry pattern variation. Different direction of asymmetry pattern across landmark for each age group in black points. Black points = asymmetry; Gray points = Mean shape of each age group.


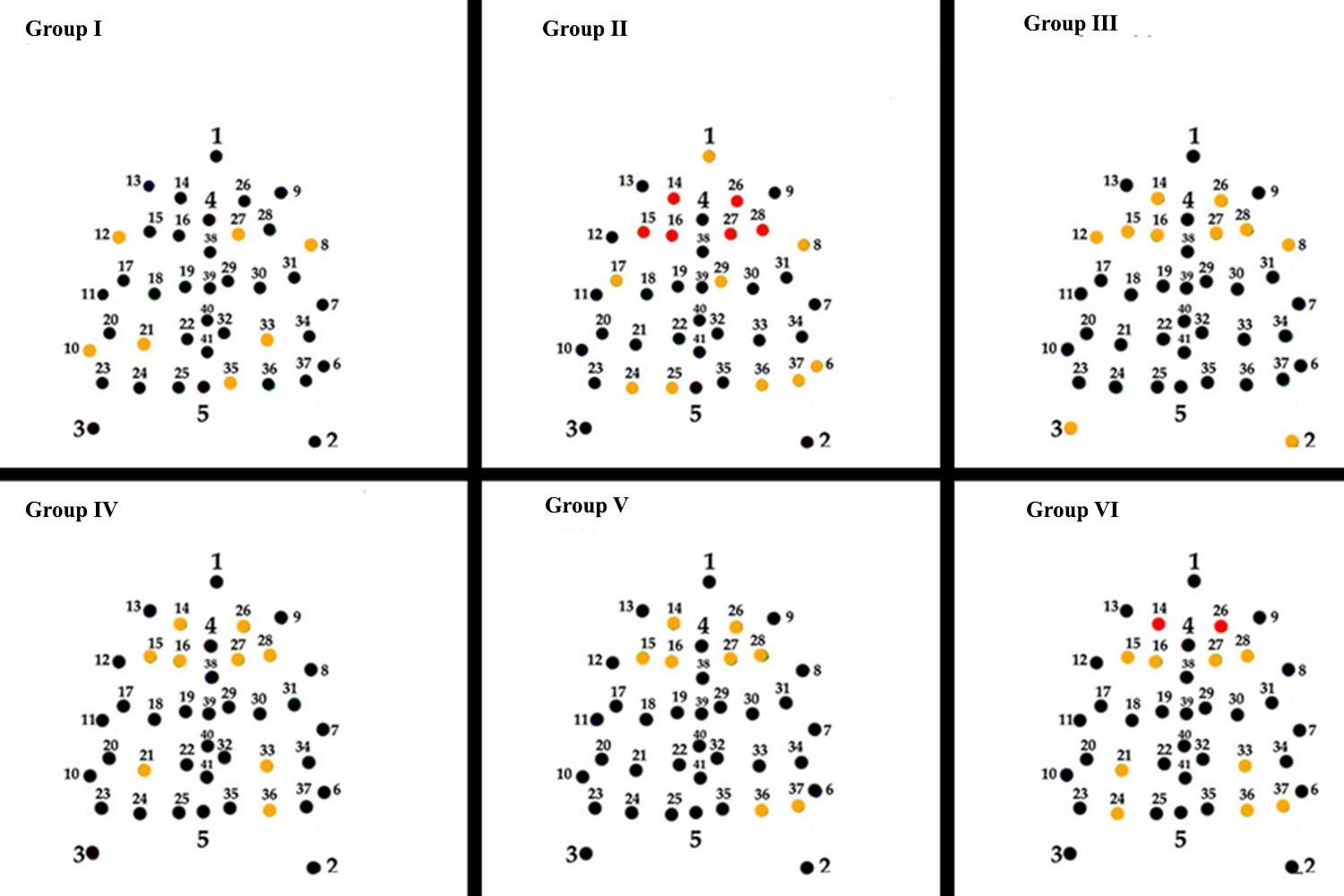


**Figure S3.** Directional Asymmetry. Values obtained for DA computed for each age group as the Procrustes distance between the mean of all original palatal shape configurations and the mean of all the reflected and relabelled configurations. Different degree of alteration (values) has been identified by color points. Black points = from 0.000 to 0.005; orange points = from 0.0005 to 0.010; red points = from 0.010 to 0.015.

**Table S1.** Results of the Permutational Multivariate Analysis of Variance (PERMANOVA) performed to assess whether the computed distribution of Procrustes distances was significantly different between the two geographical groups (Florence and Bologna) in each age group. All the p-values are Bonferroni corrected.

|  | Df | SS | MS | Rsq | F | p-val | p-adj |
| --- | --- | --- | --- | --- | --- | --- | --- |
| gI | 1 | 0.0020118 | 0.0020118 | 0.10818 | 0.3639 | 0.8 | 1 |
| Residuals | 3 | 0.0165858 | 0.0055286 | 0.89182 |  |  |  |
| Total | 4 | 0.0185977 |  | 1.00000 |  |  |  |
| gII | 1 | 0.0010929 | 0.0010929 | 0.04149 | 0.51943 | 0.68 | 1 |
| Residuals | 12 | 0.0252473 | 0.0021039 | 0.95851 |  |  |  |
| Total | 13 | 0.0263401 |  | 1.00000 |  |  |  |
| gIII | 1 | 0.015892 | 0.015892 | 0.11308 | 1.1475 | 0.39 | 1 |
| Residuals | 9 | 0.124642 | 0.013849 | 0.88692 |  |  |  |
| Total | 10 | 0.140534 |  | 1.00000 |  |  |  |
| gIV | 1 | 0.006655 | 0.0066546 | 0.09809 | 2.6101 | 0.04 | 0.24 |
| Residuals | 24 | 0.061190 | 0.0025496 | 0.90191 |  |  |  |
| Total | 25 | 0.067845 |  | 1.00000 |  |  |  |
| gV | 1 | 0.00131 | 0.0013091 | 0.00262 | 0.21055 | 0.99 | 1 |
| Residuals | 80 | 0.49739 | 0.0062174 | 0.99738 |  |  |  |
| Total | 81 | 0.49870 |  | 1.00000 |  |  |  |
| gVI | 1 | 0.001607 | 0.0016067 | 0.0076 | 0.3293 | 0.90 | 1 |
| Residuals | 43 | 0.209803 | 0.0048791 | 0.9924 |  |  |  |
| Total | 44 | 0.211410 |  | 1.0000 |  |  |  |

Abbreviation: Df = Degrees of freedom; SS = Sums of squares; MS = Mean square; Rsq = r squared; F= Fisher value; p-val = p-values; p-adj = Bonferroni corrected p-values.

**Table S2.** Fluctuating Asymmetry. Results of the Kruskal-Wallis test computed to detect the presence of significant differences between groups in the distribution of FA values across landmarks. All the p-values are Bonferroni corrected. Significant differences shown in bold.

|  | stat | Df | p-value(Bonferroni corrected) |
| --- | --- | --- | --- |
| 1 | 1.4172 | 5 | 1 |
| 2 | 28.842 | 5 | **0.001021147** |
| 3 | 28.842 | 5 | **0.001021147** |
| 4 | 4.8023 | 5 | 1 |
| 5 | 18.864 | 5 | 0.083524392 |
| 6 | 19.736 | 5 | 0.057427877 |
| 7 | 8.4719 | 5 | 1 |
| 8 | 8.0631 | 5 | 1 |
| 9 | 4.2298 | 5 | 1 |
| 10 | 19.736 | 5 | 0.057427877 |
| 11 | 8.4719 | 5 | 1 |
| 12 | 8.0631 | 5 | 1 |
| 13 | 4.2298 | 5 | 1 |
| 14 | 4.7128 | 5 | 1 |
| 15 | 3.3917 | 5 | 1 |
| 16 | 3.8214 | 5 | 1 |
| 17 | 7.6252 | 5 | 1 |
| 18 | 6.5283 | 5 | 1 |
| 19 | 7.1746 | 5 | 1 |
| 20 | 9.9049 | 5 | 1 |
| 21 | 8.3278 | 5 | 1 |
| 22 | 11.08 | 5 | 1 |
| 23 | 4.6074 | 5 | 1 |
| 24 | 10.339 | 5 | 1 |
| 25 | 8.6812 | 5 | 1 |
| 26 | 4.7128 | 5 | 1 |
| 27 | 3.3917 | 5 | 1 |
| 28 | 3.8214 | 5 | 1 |
| 29 | 7.6252 | 5 | 1 |
| 30 | 6.5283 | 5 | 1 |
| 31 | 7.1746 | 5 | 1 |
| 32 | 9.9049 | 5 | 1 |
| 33 | 8.3278 | 5 | 1 |
| 34 | 11.08 | 5 | 1 |
| 35 | 4.6074 | 5 | 1 |
| 36 | 10.339 | 5 | 1 |
| 37 | 8.6812 | 5 | 1 |
| 38 | 4.3755 | 5 | 1 |
| 39 | 4.8533 | 5 | 1 |
| 40 | 3.3545 | 5 | 1 |
| 41 | 11.037 | 5 | 1 |

Abbreviation: stat = test statistic; Df = Degrees of freedom; p-value(Bonferroni corrected) = Bonferroni corrected p-value.
